# *Phytophthora austrocedri* Elicitates Changes in Diterpene Profile of *Austrocedrus chilensis*

**DOI:** 10.3390/molecules200815084

**Published:** 2015-08-18

**Authors:** Verónica Rachel Olate, María Laura Vélez, Alina Greslebin, Guillermo Schmeda-Hirschmann

**Affiliations:** 1 Laboratorio de Química de Productos Naturales, Instituto de Química de Recursos Naturales, Universidad de Talca, Casilla 747, 3460000 Talca, Chile; E-Mail: volate@utalca.cl; 2 CONICET-Área de Protección Forestal, Centro de Investigación y Extensión Forestal Andino Patagónico (CIEFAP), 9200 Esquel, Chubut, Argentina; E-Mail: mvelez@ciefap.org.ar; 3 CONICET-Facultad de Ciencias Naturales, Universidad Nacional de la Patagonia, 9200 Esquel, Chubut, Argentina; E-Mail: agreslebin@unpata.edu.ar

**Keywords:** *Phytophthora austrocedri*, *Austrocedrus chilensis*, resin diterpenes, Cupressaceae

## Abstract

The populations of the Andean Cupressaceae *Austrocedrus chilensis* have been severely affected by a disease caused by the phytopathogenic fungus *Phytophthora austrocedri*. A study was undertaken to disclose changes in the resin composition of *P. austrocedri*-infected individuals, including naturally infected and artificially inoculated trees, compared with healthy *A. chilensis* trees. GC-MS and ^1^H-NMR studies showed a clear differentiation among healthy and infected resins, with the diterpene isopimara-8(9),15-dien-19-ol as a relevant constituent in resins from infected trees. The effect of resin fractions from *P*. *austrocedri* infected trees on the pathogen was assessed by measuring the mycelial growth in agar plates. The most active fractions from resin obtained from infected trees inhibited fungal growth by nearly 50% at 1 mg/dish (35.37 µg/cm^2^). The main constituent in the active fractions were 18-hydroxymanool and the aldehyde torulosal. Both compounds are oxidation products of manool and can be a chemical response of the tree to the pathogen or be formed from the pathogen as a biotransformation product of manool by microbial oxidation. While the diterpene profiles from *A. chilensis* tree resins can easily differentiate healthy and *P*. *austrocedri* infected individuals, the possible conversion of manool to the antifungal derivatives 4 and 6 by the microorganism remains to be established.

## 1. Introduction

The exudation of resins, a mixture of monoterpenes, sesquiterpenes, and diterpene resin acids, is a major part of the constitutive defense mechanisms in conifers. It has been reported that the resins protect against pathogenic agents and insects among other types of damage [1,2,3,4]. Resin acts by blocking wounds, repelling or flushing invader organisms out of the bark, and entrapping organisms by its sticky nature, inhibiting or even killing the invader due to its toxicity.

Resin can be also part of the inducible defense mechanisms, as in the case of traumatic resin ducts that are created *de novo* in response to wounds, insect damage or pathogenic fungi. The resin formed by traumatic ducts can be different than constitutive resin [5,6,7], and may be more toxic through changes in terpenoid components or addition of phenolics [7].

Whereas constitutive chemical defenses are generally non-selective with respect to the invader organism, inducible chemical defenses include both broad-spectrum and specific compounds. The resin of conifers contains mainly diterpenes [8,9,10]. Diterpenes in resins have been studied by NMR [11,12,13] and GC-MS [10,14,15]. The variability of the resin terpene composition in conifer species was investigated by Cool *et al*. (1991) [16] and Cool (1996) [17] in *Fitzroya cupressoides.* The diterpene composition of the conifer *Austrocedrus chilensis* was reported by Olate *et al*. (2011) [18] and seasonal variation in resin composition was also described [19]. There is no information about the variability on diterpene composition in this species under biotic or abiotic stress.

In the last few decades, the Cupressaceae *Austrocedrus chilensis*, known as “ciprés de la cordillera”, has suffered a devastating disease known as “*mal del ciprés*” or, recently, as “*Austrocedrus chilensis root disease”* [20,21,22]. This disease is characterized by chlorosis, withering of the foliage, progressive defoliation and the death of the tree. It is caused by the pathogen *Phytophthora austrocedri* (Oomycota, Straminipila) [20,21,22].

There are few studies on antifungal activity of resins against *Phytophthora* spp. [23,24] in spite of the fact that it is a frequent response of trees to *Phytophthora* infection [25,26,27,28,29]. *A. chilensis* profusely produces resin associated with *Phytophthora* lesions in phloem. Resin-pockets are usually developed below bark in the advance of the *Phytophthora* lesion [22]. Whether this resin is a specific response to pathogen infection or simply a structural unspecific response is still unknown.

To elucidate this aspect, the composition of *A. chilensis* resin and its antifungal activity was investigated. The hypothesis was that the resin composition differs on healthy and *Phytophthora*-diseased trees. To test this hypothesis, resin profile from healthy, naturally infected and artificially infected trees was compared. Fractionated resin of affected trees was tested for antifungal activities.

The present work includes the profiling of resin from healthy and diseased trees of *Austrocedrus chilensis* and reports for the first time the effect of the *A. chilensis* resin on the growth of *P. austrocedri*.

## 2. Results and Discussion

### 2.1. Antifungal Activity

**Figure 1:**
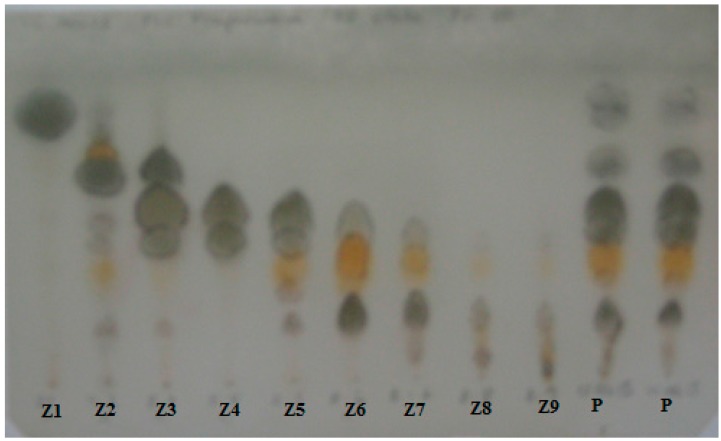
TLC analysis of the resin from *A. chilensis* tree naturally infected with *P. austrocedri* (P) (AR-15 sample) and the preparative TLC fractions Z1-Z9. Spots were revealed by spraying with *p*-anisaldehyde and heating. Silica gel, PE:EtOAc 8:2 (*v*:*v*).

**Figure 2:**
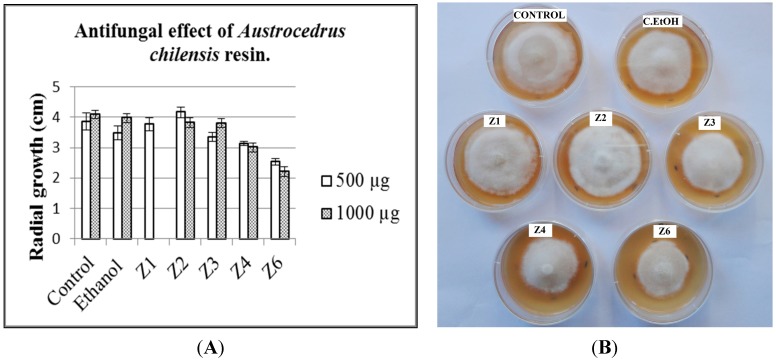
Antifungal effect of *A. chilensis* resin on *P. austrocedri*. (**A**) Graphic of the radial growth in agar dishes; (**B**) Growth inhibition assays for fungal growth. 500 µg of each fraction were distributed in the dish giving a concentration of 17.68 µg sample/cm^2^. The controls are presented in the top of the image. Control: Untreated *P. austrocedri*; C. EtOH: Ethanol control. Z1–Z6: Tested resin fractions.

The resin samples were compared by thin layer chromatography (TLC) to disclose similarities and differences among the different variables (healthy, natural infection and artificially inoculated trees). A representative resin from naturally infected trees (sample AR-15, Table 1) was selected for fractionation and antifungal testing. Preparative TLC afforded nine fractions, with most of the compounds eluting in the fractions Z1 to Z6 (Figure 1). A comparison of the means of fungal growth after 14 days inoculation is presented in Figure 2A. Growth inhibition assay of the different fractions was assessed by the radial growth of the fungus at 0.5 and 1 mg of the fractions per dish, corresponding to 17.68 and 35.37 µg sample/cm^2^ (Figure 2B). At 1 mg per dish (35.37 µg sample/cm^2^), the most active fractions Z4 and Z6 inhibited fungal growth by 26.4% and 46.2%, respectively while the effect at 0.5 mg (17.68 µg sample/cm^2^) was 18.9% and 34.2%, respectively. Fractions Z4 and Z6 significantly reduced *P. austrocedri* growth (*p* < 0.001 *vs.* ethanol control) (Figure 2). Fraction Z3 inhibited fungal growth by 13.2% and 7.1% at 0.5 (17.68 µg sample/cm^2^) and 1 mg/dish (35.37 µg sample/cm^2^), respectively.

**Table 1:** Resin sample collection (November, 2011). Río Grande Valley, Parque Nacional Los Alerces, Provincia del Chubut, Argentina. F: Female, M: Male, U: Unknown.

Tree	Gender	Sample Number	Observations
A03	F	AR-01	Artificially inoculated
A07	F	AR-02, AR-03, AR-04	Artificially inoculated
A08	M	AR-05, AR-06	Healthy and artificially infected zones
A09	F	AR-07, AR-08, AR-09	Artificially inoculated
A06	F	AR-10	Artificially inoculated
A18	U	AR-11, AR-12	Artificially inoculated
A21	U	AR-13	Artificially inoculated
I01	U	AR-14	Infected, natural lesion
I02	U	AR-15	Infected, natural lesion *
S01	F	AR-16	Healthy tree, naturally exuded resin
S02	F	AR-17	Healthy tree, naturally exuded resin
S03	F	AR-18	Healthy tree, naturally exuded resin
S04	F	AR-19	Healthy tree, naturally exuded resin
S05	M	AR-20	Healthy tree, naturally exuded resin
S06	M	AR-21	Healthy tree, naturally exuded resin
S07	M	AR-22	Healthy tree, naturally exuded resin
S08	M	AR-23	Healthy tree, naturally exuded resin
S09	M	AR-24	Healthy tree, naturally exuded resin

* Sample used for the antifungal activity study.

### 2.2. Resin Profiling by GC-MS and ^1^H-NMR

**Figure 3:**
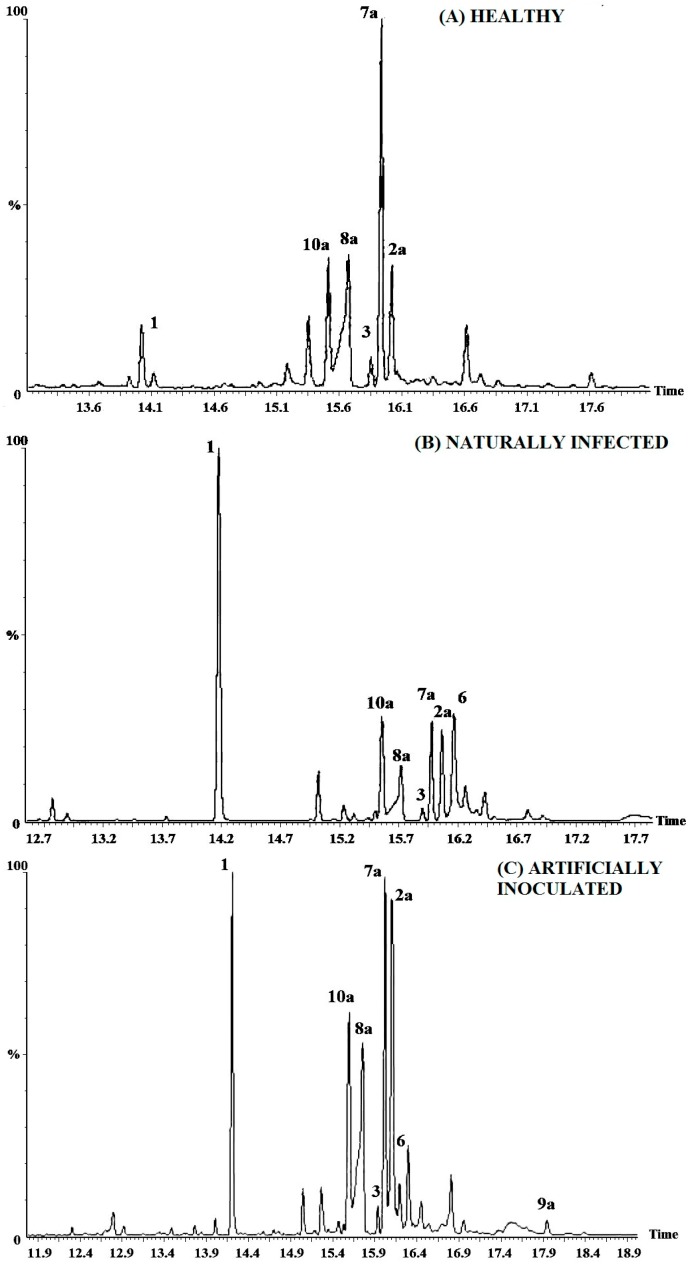
GC profiles of the *Austrocedrus chilensis* resin constituents from healthy and *Phytophthora austrocedri*-infected trees. (**A**) Healthy; (**B**) Naturally infected; (**C**) Artificially inoculated.

**Figure 4:**
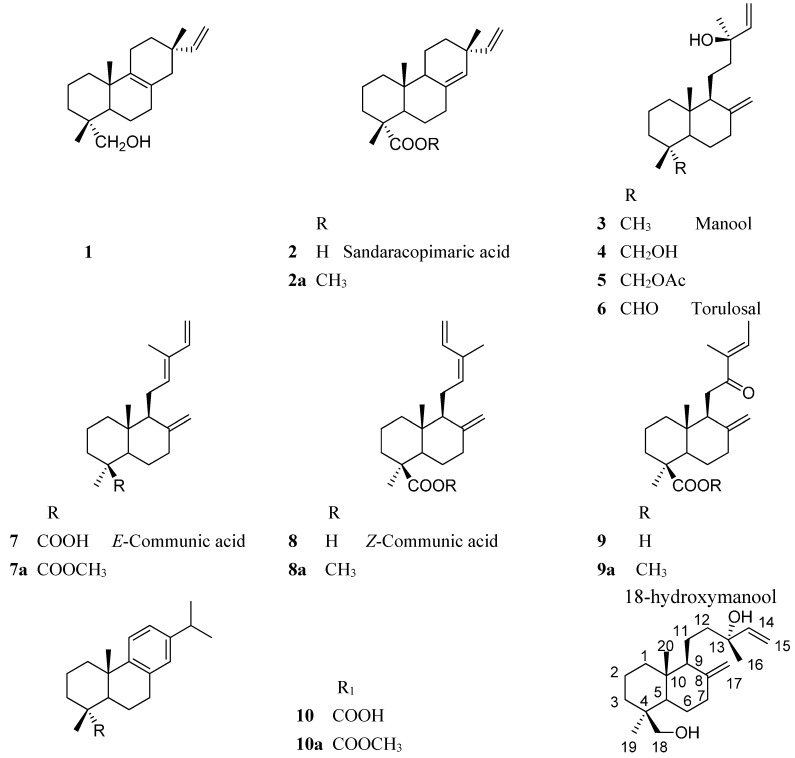
Structure of the compounds isolated and/or identified from the resin fractions of *Austrocedrus chilensis* trees naturally infected with *Phytophthora austrocedri* inhibiting radial growth of the phytopathogenic fungus.

The different resin samples, including healthy, naturally infected and *P. austrocedri*-inoculated male and female trees were compared by TLC (Figure 1), GC-MS (Figure 3) and ^1^H-NMR. Figure 4 is showing the structures of compounds identified in resin from *A. chilensis* trees. The resin profiles from healthy trees were similar to those described by Olate *et al*. (2014) [19] for female and male individuals from a Chilean population of *A. chilensis*. However, naturally and artificially infected trees showed a distinctive and different profile with relevant changes in chemical composition. A peak at Rt 14.17 min was diagnostic for the infected resins. The mass fragmentation pattern of the compound is in agreement with the diterpene isopimara-8(9),15-dien-19-ol **1**. Representative GC chromatograms of a healthy tree, a naturally infected and an artificially inoculated individual are shown in Figure 3.

The resin fractions showing inhibitory effect on the growth of *P. austrocedri* were analyzed by GC-MS and ^1^H-NMR to identify the constituents. In the GC chromatogram of Z3, two main compounds were observed, eluting at Rt = 14.17 and 15.82 min. The ^1^H-NMR analysis of the fraction showed the two main constituents, both sharing a common –CH=CH_2_ and differing in the presence of a primary alcohol for the main compound and an exomethylene for the second diterpene. The spectra are in agreement with the diterpene **1** and manool **3**, previously isolated from the tree resin [18]. The assignation of the diagnostic H signals is listed below.

Compound (*Isopimara-8(9),15-dien-19-ol*) (**1**): 5.77 dd (17.5, 10.8 Hz, H-15), 4.91 dd (11.0, 1.7, 1H, H-16); 4.84 dd (17.5, 1.1, 1H, H-16ʹ), 3.45 d (10.9, 1H, H-19); 3.18 d (10.9, 1H, H-19ʹ), 1.01 s (3H, H-17), 0.98 s (3H, H-18), 0.82 s (3H, H-20).

Compound (*Manool*) (**3**): 5.92 dd (*J* = 17.3, 10.7, 1H, H-14); 5.22 dd (17.4, 1.1, 1H, H-15); 5.06 dd (10.7, 1.1, 1H, H-15ʹ), 4.83 d (1.3, 1H, H-17); 4.53 brs (1H, H-17ʹ), 1.29 s (3H, H-16), 0.88 s (3H, H-19), 0.82 s (3H, H-20).

The fraction Z4-5 showed in the ^1^H-NMR spectrum an aldehyde H at δ 9.67 ppm, assignable to the main constituent as well as the –CH=CH_2_ side chain, an exomethylene and three methyl singlets at δ 1.20, 0.94 and 0.49 ppm, compatible with torulosal **6**. Several minor compounds also occurs in the fraction, including the isomers *E*-communic acid **7** and *Z*-communic acid **8** as well as the minor components **1**, **5**, and **9**, identified by comparison with literature [18]. GC-MS analysis showed a main compound at Rt = 16.16 min., identified as torulosal **6**, *E*-communic acid methyl ester **7a** (Rt = 15.97 min, *m*/*z* = 316); *Z*-communic acid methyl ester **8a** (Rt = 15.71 min, *m*/*z* = 316) and 12-oxolabda-8(17), 13*E*-dien-19 oic acid methyl ester **9a** (Rt = 17.89 min, *m*/*z* = 332), all of them in agreement with the literature [18,19].

The fraction Z6-7 presented in the GC-MS chromatogram a main compound (>90%) at Rt = 17.13 min. With a molpeak of *m*/*z* 288, in agreement with the consecutive loss of two molecules of water from the diterpene 8(17),14-labdadien-13,18-diol (18-hydroxymanool) **4**, reported from *A. chilensis* resin [18]. Two additional minor compounds were detected and identified as dehydroabietic acid methyl ester **10a** (Rt = 15.54 min, *m*/*z* = 316) and sandaracopimaric acid methyl ester **2a** (Rt = 16.06 min, *m*/*z* = 316). The ^1^H-NMR spectrum of the fraction showed a main constituent with a monosubstituted double bond (5.91 dd, *J* = 17.3, 10.7; 5.20 dd, *J* = 17.4, 1.1; 5.05 dd, *J* = 10.8, 1.1 Hz), a exomethylene (δ 4.82 s, 4.52 s) and a primary alcohol (3.76 d, *J* = 10.9 and 3.38 d, 10.9 Hz). Three methyl singlets at δ 1.28, 0.98 and 0.65 ppm further support the assignation of a diterpene skeleton with a tertiary alcohol function, in agreement with 18-hydroxymanool **4**. The diagnostic ^1^H-NMR signals and the ^13^C-NMR spectral data of the compound are listed below.

Compound (*18-Hydroxymanool*) (**4**): ^1^H-NMR (400 MHz, CDCl_3_, δ-values, *J* in Hz): 5.91 dd (*J* = 17.3, 10.7, 1H, H-14); 5.20 dd (17.4, 1.1, 1H, H-15); 5.05 dd (10.8, 1.1, 1H, H-15’), 4.82 s (1H, H-17); 4.52 s (1H, H-17ʹ), 3.76 d (10.9, 1H, H-18); 3.38 d (10.9, 1H, H-18ʹ), 1.28 s (3H, H-16), 0.98 s (3H, H-19), 0.65 s (3H, H-20). ^13^C-NMR (100 MHz, CDCl_3_, δ-values): 148.10 s, 145.19 d, 111.61 t, 106.78 t, 73.59 s, 64.70 t, 57.40 d, 56.34 d, 41.31 t, 39.73, 38.97, 38.81, 38.63, 35.40, 27.47, 27.15, 24.39, 18.98, 17.81, 15.27 q. ROESY experiments shows correlation of the methyl group at δ 0.64 with the –CH_2_OH protons at δ 3.34 and 3.72 ppm.

**Figure 5:**
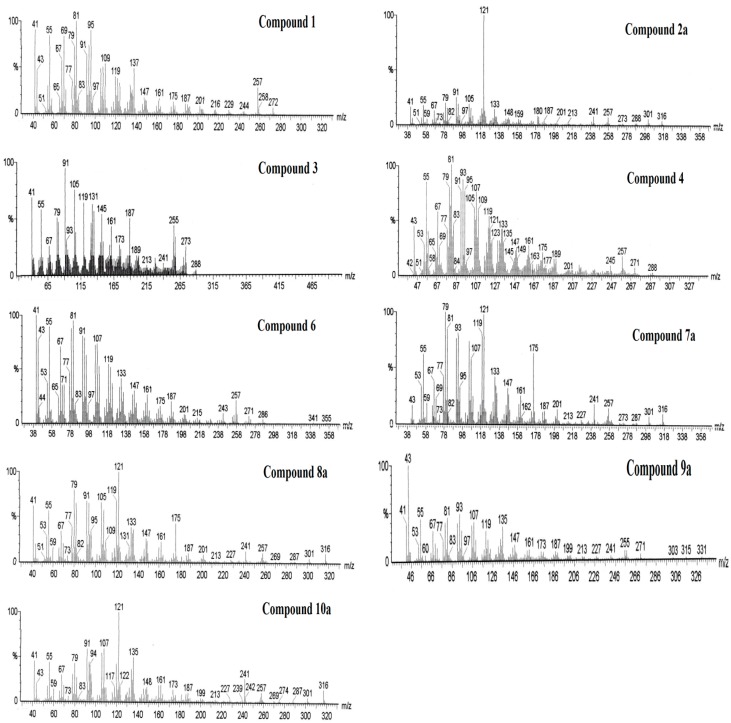
Mass spectra of the compounds **1**, **2a**, **3**, **4**, **6**, **7a**, **8a**, **9a** and **10a** identified from the resin fractions of *Austrocedrus chilensis* trees inhibiting radial growth of the phytopathogenic oomycete *Phytophthora austrocedri*.

The fractions Z3, Z4-5 and Z6-7, obtained by preparative TLC of the resin from *A. chilensis* trees infected with *P. austrocedri* accounts for 15.0%, 28.3% and 19.4%, respectively, from the total resin constituents. The most active fraction Z6-7 contains mainly 18-hydroxymanool **4** with **2** and **10** as minor constituents. Fractions Z4-5 yielded the aldehyde torulosal **6** as main compound with **1**, **5**, **7**–**9** as additional minor components. The less active fraction Z3 contained **1** as main compound, the alcohol **3** and the aldehyde **6** as other constituents. Mass spectra of the compounds **1**, **2a**, **3**, **4**, **6**, **7a**, **8a**, **9a** and **10a** are shown in Figure 5. The fragmentation patterns were described in a previous work [18].

Production of differential resins in response to wounds, insect attack or fungal invasion has been reported [6,7,30], as well as the antifungal activity of these “defense-response resins” [2]. A four-fold increase of diterpenoids content was found in induced resin of inner bark of *Picea* spp. [6,7], the highest increment was observed in manoyl oxide [7].

Antifungal activity in conifers has been reported in *Taxodium distichum* cones, which contain mainly abietane-type diterpenes [31]. Sandaracopimarinol and ferruginol from extracts of *Cryptomeria japonica* were considered the most active constituents against phytopathogenic microorganisms (*Fusarium oxysporum*, *Phytophthora capsici*, *Phytium splendens* and *Ralstonia solanacearum*) [32]. Inhibitory action of volatile components of *Pinus* spp. oleoresins on *Phytophthora* spp. has been shown by Bunny and Tippet (1988) [23]. Previous work on induced resins have demonstrated that this resin with differential composition is produced *de novo* in traumatic resin ducts formed as a response to injury [5,6]. Thus, it would be of interest to investigate the possible presence of this kind of ducts in *A. chilensis* tissues.

The chemical composition of resins from healthy *A. chilensis* trees compared with that of *P. austrocedri*-infected individuals, including naturally infected and artificially inoculated trees showed a clear difference (Figure 3), with a diagnostic peak of compound **1** for the resins of infected trees. The fraction containing this compound as main constituent showed a slight inhibitory growth effect on *P. austrocedri*. The main component of the most active resin fraction Z6-7, is the C-18 oxidized product of manool, 18-hydroxymanool **4**. The fraction inhibited growth of *P. austrocedri* by 34.2% at 0.5 mg/dish (17.68 µg sample/cm^2^) and by 46.2% at 1 mg/dish (35.37 µg sample/cm^2^), respectively. Manool was reported as having toxicity towards the brine shrimp and cytotoxic effects [33]. The compound showed antibacterial activity against *Staphylococcus aureus* and *Stenotrophomonas maltophilia*, antifungal effect against *Candida albicans* and cytostatic activity on human cancer cells [34]. The role of this compound on the defense strategy of *A. chilensis* to *P. austrocedri* infection deserves further studies.

The compound **4** can be further oxidized to the aldehyde **6** (torulosal) found as the major constituent of the Z4-5 fraction. The results suggest that different oxidation products from the diterpene manool play a role in the antifungal effect of the resin. Further studies should be carried out to disclose if the oxidation of manool is a host response or if the derivatives are formed via microbial oxidation (biotransformation) of the parent diterpene. Aranda *et al*. (1999) [35] reported the oxidation of manool by *Mucor plumbeus* to the 18-hydroxy derivative as well as several product of fungal bioconversion of labdane diterpenes. A comprehensive review of biotransformations of di-, triterpenes, steroids and other natural products has been summarized by Asakawa and Noma (2010) [36]. While the chemical profiles from *A. chilensis* tree resins can easily differentiate healthy and *P*. *austrocedri* infected individuals, the possible convertion of manool to the antifungal derivatives **4** and **6** by the microorganism remain to be established.

## 3. Experimental Section

### 3.1. Plant Material

**Figure 6:**
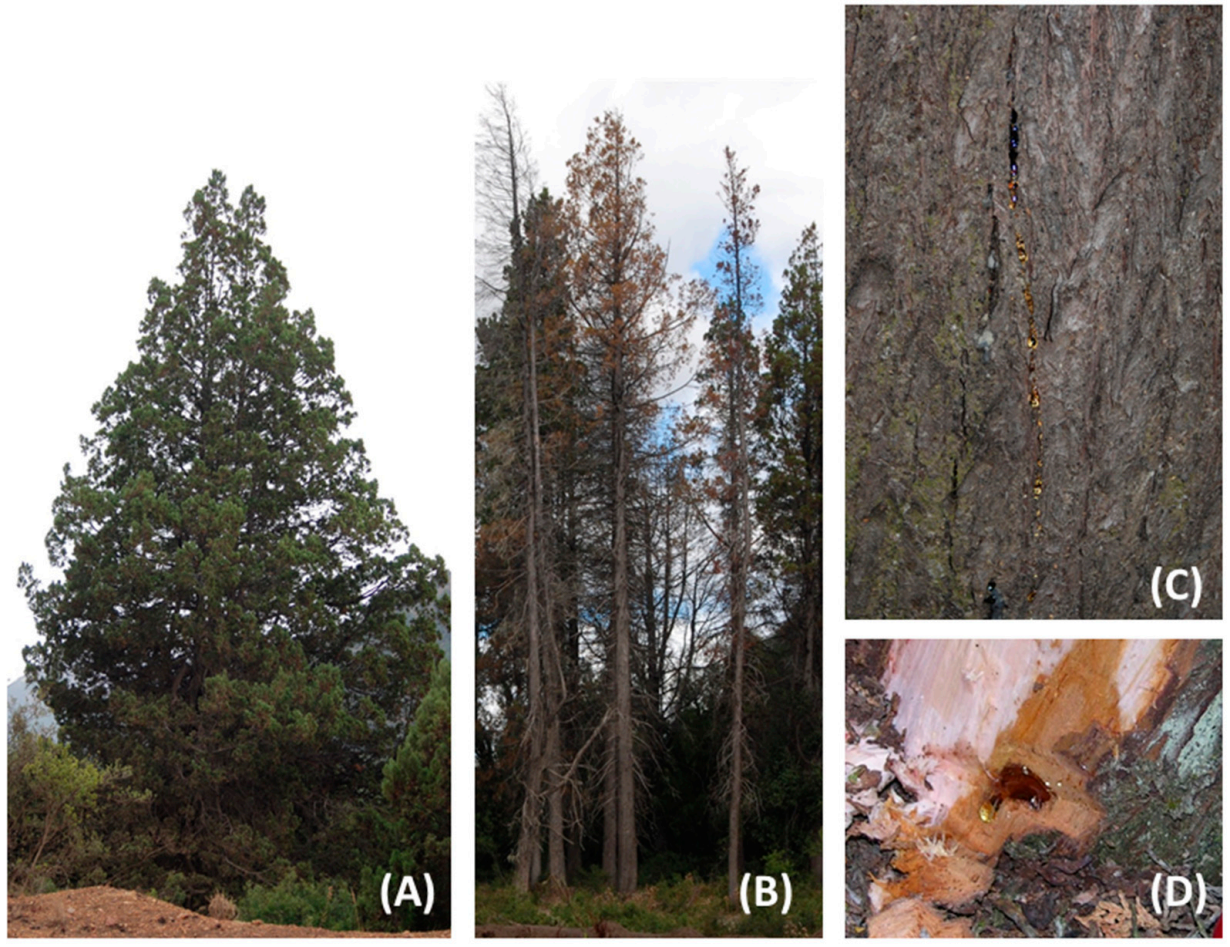
(**A**) Healthy *Austrocedrus chilensis* tree showing no symptoms of *Phytophthora austrocedri* infection; (**B**) Diseased *Austrocedrus chilensis* tree infected with *Phytophthora austrocedri*; (**C**) Resin exudates on bark of the tree base; (**D**) Detail of a resin pocket associated to a *P. austrocedri* lesion. Source: (**A**–**C**): Alina Greslebin; (**D**): Everett M. Hansen.

Resin from phloem of male and female *A. chilensis* trees, including healthy and *P. austrocedri*-diseased trees was collected. To further confirm possible changes in resin composition associated with the fungal infection, resin from artificially inoculated *P. austrocedri* individuals (controlled conditions) were also included in the study. Trees were located in the Los Alerces National Park and the 16 de Octubre Valley, Chubut Province, Argentina (Figure 6). The resins were stored in glass containers, hermetically closed until analysis. The origin, sex and observations on the samples are summarized in Table 1.

### 3.2. General Procedures

All solvents used in chromatographical procedures were previously distilled. Pre-coated silica gel plates (Merck, Darmstadt, Germany, Kieselgel 60 F 254, 0.25 mm) were used for TLC. TLC spots were visualized by spraying the chromatograms with *p*-anisaldehyde/ethanol/acetic acid/H_2_SO_4_ (2:170:20:10 *v*/*v*) and heating at 110 °C for 3 min.

### 3.3. Resin Analysis by TLC and Isolation of the Resin Constituents

The composition of the different resin samples was first assessed by analytical TLC using silica gel as stationary phase and PE:EtOAc 80:20 (*v*/*v*) as eluent. The spots were visualized under UV light and revealed with *p*-anisaldehyde reagent and heating until development of colors. After TLC comparison, representative samples of the resin of male and female trees, including healthy, naturally infected and inoculated individuals were selected for GC-MS and NMR studies.

A representative sample from a diseased individual was selected for the preparative isolation of the constituents and antifungal-guided identification of the resin bioactives. The sample (400 mg) was separated by preparative TLC (silica gel, PE:EtOAc 80:20 *v*/*v*) in nine different zones, according to the Rf values and visualization under UV light. The bands were scraped off, extracted with ethyl acetate, filtered and taken to dryness to afford the fraction Z1 to Z9. Fractions Z4 and Z5 showing a similar composition were pooled as well as fractions Z6 and Z7. Fractions Z8 and Z9 contained mainly degraded products and were not further investigated. The fractions were assessed for activity against *P. austrocedri* and analyzed by GC-MS (as methyl esters) and ^1^H-NMR. The compounds/fractions were stored in closed glass containers until the antifungal assays.

### 3.4. Antifungal Activity Assays

The fractions obtained by preparative TLC of the sample were evaluated as antifungal agents against *Phytophthora austrocedri*. The assays were performed with 0.5 and 1.0 mg fraction per Petri dish (60 mm, surface area: 28.27 cm^2^) using tomato agar as culture medium. The obtained fractions were dissolved in 25 µL ethanol (Molecular Biology grade) and spread in the surface of sterile dishes containing the culture medium. The liquid was distributed uniformly with a triangular handle. The Petri dish surface was 28.27 cm^2^. Therefore, the 500 and 1000 µg/dish are about 17.68 and 35.37 µg fraction/cm^2^, respectively. After drying the plates, a 5 mm plug from the margin of an actively growing colony of *Phytophthora austrocedri* was fixed in the central part of the agar. The plates were sealed and incubated at 15 °C in the dark. The radial growth of the pathogen was measured after 14 days. Treatments and the respective controls (solvent control: ethanol and untreated control) were done by quintuplicated. ANOVA was used to compare mean growth rates. *Post hoc* Tukey test were used for multiple comparisons of groups according to normality and homogenity of variance based on the Levene test (SPSS Statistics 17.0).

### 3.5. GC-MS Analysis

The resin samples were dissolved in anhydrous chloroform and treated with diazomethane in diethyl ether to obtain the methyl esters of carboxylic acids for analysis. The derivatized resins were dissolved in dichloromethane and 1 µL was injected in the GC-MS apparatus for analysis. The method used was described in Olate *et al*. (2014) [19]. Equipment: Perkin Elmer Turbo Mass (Perkin-Elmer Corporation, Norwalk, CT, USA). Column: fused silica capillary column, SP-2330 (Supelco, St. Louis, MO, USA), 30 m × 0.25 μm. Carrier: He, split flow 50.0 mL/min, initial set point: 20.0 PSIG. Oven program: total run time: 66 min, initial temperature: 100 °C, initial hold: 1.00 min, Ramp: 10.0 °C/min to 250 °C, hold for 50.00 min. Injection volume: 1 μL. Compounds were characterized by electron-ionization (EI) mass spectra. Retention time (Rt, min) and MS fragmentation patterns of the known compounds were compared with literature (Olate *et al*., 2011, 2014) [18,19].

### 3.6. ^1^H-NMR Analysis

All NMR experiments were performed on a Bruker Avance 400 NMR spectrometer (Bruker BioSpin GmbH, Rheinstetten, Germany) equipped with a 5 mm inverse detection z-gradient probe. The ^1^H spectra were measured at 400 MHz at room temperature (20 °C) using CDCl_3_ as solvent. Chemical shifts are given on the δ scale and were referenced to residual CHCl_3_ at 7.25 ppm. One-dimensional ^1^H spectra were acquired under standard conditions. Additional experiments for structural elucidation included ^13^C-NMR, Distortionless Enhancement of NMR signals by Polarization Transfer (DEPT), Rotating frame Overhauser Effect Spectroscopy (ROESY) for the main compound from the fraction most active against *P. austrocedri*.

## 4. Conclusions

In this work, the changes in the diterpene composition of *A. chilensis* resin in the presence of the phytopathogenic fungus *Phytophthora austrocedri* were studied. The diterpene composition of resins from naturally and artificially infected trees was compared with resins from healthy individuals. The resin profiles were studied by GC-MS and H-NMR techniques. Resins from *P. austrocedri* infected trees showed relevant differences in its diterpene profiles in comparison with resin from healthy trees. The effect of resin from *P. austrocedri* infected trees on the growth of this pathogen was evaluated *in vitro*, using different resin fractions obtained by chromatographic means. The resin from *A. chilensis* taken from an infected tree exposed an interesting antifungal effect evaluated as the inhibition of mycelial growth in agar plates. The most active resin fractions contained 18-hydroxymanool and torulosal as the main constituents. These compounds are oxidation products of manool, probably produced by the tree as a chemical response against the fungal infection or formed by the pathogen as a biotransformation product.

The differences in the diterpene resin composition due to *P. austrocedri* infection were established in this work. However, the possible microbial conversion of manool to its oxidized derivatives requires further investigation.
